# Towards defining the core *Saccharum* microbiome: input from five genotypes

**DOI:** 10.1186/s12866-022-02598-8

**Published:** 2022-08-08

**Authors:** Juliane K. Ishida, Andressa P. Bini, Silvana Creste, Marie-Anne Van Sluys

**Affiliations:** 1 Departamento de Botânica, Instituto de Biociências, Universidade de São Paulo, Rua do Matão 277, Cidade Universitária, São Paulo, SP 05508-090 Brazil; 2 Present address: Departamento de Botânica, Instituto de Ciências Biológicas, Universidade Federal de Minas Gerais, Av. Pres. Antônio Carlos, 6627 - Pampulha, Belo Horizonte, MG 31270-901 Brazil; 3 Centro de Cana, IAC-Apta, Ribeirão Preto, Av. Pádua Dias n11, CEP 13418-900, Piracicaba, São Paulo, Brazil

**Keywords:** V3-V4 rDNA, Phyllosphere, Rhizosphere, Sugarcane, Plant tissues, Soil microbiota

## Abstract

**Background:**

Plant microbiome and its manipulation inaugurate a new era for plant biotechnology with the potential to benefit sustainable crop production. Here, we used the large-scale 16S rDNA sequencing analysis to unravel the dynamic, structure, and composition of exophytic and endophytic microbial communities in two hybrid commercial cultivars of sugarcane (R570 and SP80–3280), two cultivated genotypes (*Saccharum officinarum* and *Saccharum barberi*) and one wild species (*Saccharum spontaneum*).

**Results:**

Our analysis identified 1372 amplicon sequence variants (ASVs). The microbial communities’ profiles are grouped by two, root and bulk soils and stem and leave when these four components are compared. However, PCoA-based data supports that endophytes and epiphytes communities form distinct groups, revealing an active host-derived mechanism to select the resident microbiota. A strong genotype-influence on the assembly of microbial communities in *Saccharum* ssp. is documented. A total of 220 ASVs persisted across plant cultivars and species. The ubiquitous bacteria are two potential beneficial bacteria, *Acinetobacter* ssp., and *Serratia symbiotica*.

**Conclusions:**

The results presented support the existence of common and cultivar-specific ASVs in two commercial hybrids, two cultivated canes and one species of *Saccharum* across tissues (leaves, stems, and roots). Also, evidence is provided that under the experimental conditions described here, each genotype bears its microbial community with little impact from the soil conditions, except in the root system. It remains to be demonstrated which aspect, genotype, environment or both, has the most significant impact on the microbial selection in sugarcane fields.

**Supplementary Information:**

The online version contains supplementary material available at 10.1186/s12866-022-02598-8.

## Background

In their natural environment, plants shelter many prokaryotes and eukaryotes whereby the intimate relationship between the host and its phytobiome impacts growth and development. Advanced sequencing techniques revealed that the diversity of microorganisms inhabiting inside the plant (endophytic) and in the zones surrounding the leaf (phyllosphere) [1] and root (rhizosphere) [2] tissues can exert a relevant biological role much like the human gut microbiota [3]. Plant interaction with beneficial microbes can trigger a systemic defense response, protecting the host plant against different pathogens [4–7], or may increase the plant tolerance upon abiotic stress [8–10], enhance metal absorption [11], or nutrient acquisition [12, 13], affecting plant physiology [14] and plant growth and development [2, 15, 16].

Sugarcane (*Saccharum* spp.) is a globally important crop. It provides most of the sugar production globally and is the most efficient bioenergy alternative to fossil fuel [17]. However, to increase agricultural productivity with minimal environmental impact, it is mandatory a constant renewal of biotechnological strategies aimed at a sustainable production system. Studies focus on beneficial services provided by microbial communities that offer alternatives to improve agronomic practices that are friendlier to the environment, intending at reducing dependence on agrochemicals. Therefore, recent studies reveal that the plant microbiome plays a relevant role in the sustainability of agriculture practices.

The challenge relies on the identification and a better understanding of the complex interaction among the biotic and abiotic factors capable of interfering in the dynamics of the ecosystem towards enhancing the microbial biological functions. Among these factors are the environmental influence and host genotypes. In this context, identifying a set of microorganisms that maintains the structure across different tissues and genetic backgrounds is the first step in optimizing the plant-microbe partnership. This core microbiome that suffers minor variation independently of external factors is responsible for performing a biological function for the host or the organisms in the surroundings [18, 19]. This research aims to determine the colonization ab initio of five *Saccharum* genotypes using non-agronomical soil. A microbiome approach compares different tissues and both endo and exophytic communities. We aim to define the core microorganisms that are associated with hybrid sugarcane cultivars and three *Saccharum* species. These organisms may represent a standard microbial set that partners plants with potential relevance to plant growth, development, and health.

## Results

### Estimating mitochondria and chloroplast contamination

The sugarcane microbiome was evaluated by collecting epiphytes and endophytes from five genotypes: a wild species (*Saccharum spontaneum*; IN-8458) two cultivated canes (*Saccharum barberi*; Chunee and *Saccharum officinarum*; Badilla), and two sugarcane hybrid commercial cultivars (R570 and SP80–3280) (Supplemental Fig. S1 - Additional file 1). Samples were collected from three-month-old plants after budding (Supplemental Fig. S2 - Additional file 1), along with the nonplanted (bulk) soil. The 16S amplicon sequencing of the root, stem, and leaves from *Saccharum* varieties and the bulk soil samples yielded a total of 5.6 billion bases distributed in 18.7 million reads. To evaluate the performance of the usage of PNA clamps to avoid the host sequence contamination we mapped the quality-trimmed reads against the mitochondria and chloroplast genomes (Supplementary Table S1 - Additional file 2). The most significant contaminant reads were detected in the endophytic compartments corresponding to the organellar ribosomal regions (Supplementary Table S1 Additional file 2). In photosynthetic tissues, contamination was 12.2% in stem and 18% in leaf tissues (Supplementary Table S1 - Additional file 2), whereas in root tissues it dropped to 0.1%. The variation is expected since the number of plastids varies among the plant compartments. There is more chloroplast in leaves than in stem and much less in roots [20]. When considering the two mitochondrial genomes of sugarcane [21], the percentage of reads is much higher than chloroplast. It corresponds to 78% in the stem, 53.2% in leaves, and 6% in the root. (Supplementary Table S1 - Additional file 2), indicating the variation of efficiency of PNA clamps to remove the mitochondrial sequences. A previous study showed that there was a remaining 20% of chloroplast contamination in photosynthetic samples even with the addition of PNA clamps in the 16S rDNA amplification reaction, with a lower efficiency observed in blocking mitochondrial sequences’ amplification in stem and leaves [22]. In root tissues, the amplification of the mitochondrial reads was maintained at around 5% when the PNA clamp is added [22]. Thus, the higher percentage of mitochondrial reads from leaves and stems, but not on roots, detected in this previous study is in good alignment with our reported data. The number of mitochondria and plastids per cell is very dynamic and changes according to the developmental stage of each tissue. A rough estimation in mesophyll cells has per-cell mitochondria ranging from ~ 200 to ~ 600 [23], compared to an average of ~ 50 chloroplasts [24]. Thus, the PNA-clamp depletion efficiency discrepancy may occur because of such differences among the photosynthetic plant cells. We estimated that the low number of organelle sequences in roots might occur due to the large aerenchyma in the sugarcane root system formed after massive programmed cell death, with few remaining living cells [25].

### *Saccharum* 16S rDNA sequencing

DADA2 denoise software [26] was used to filter ambiguity at nucleotide level, and further analyses were performed with the remainder average of 37.8% (range 34 – 43.3%) of the original data (Supplementary Table S2 - Additional file 2). The passed-filter sequences were merged and only the non-chimeric ones that correspond to about 29.7% (range of 21.6 - 37.7%) of reliable sequences (Supplementary Table S2- Additional file 2) passed to the subsequent analysis. In absolute numbers, the denoise pipeline yielded 2,960,290 paired-end sequences distributed in 1372 different amplicon sequence variants (ASVs). The number of identified ASVs is significantly lower than in previous sugarcane studies, where 23,811 operational taxonomic units (OTU) were recovered from external and internal niches of SP80–3280 [22]. About 7198 OTUs were detected in the inner root compartment of four species and two hybrid cultivars [27]. This difference mainly occurred because of the adoption of a distinct methodology. In previous studies, microbial richness was estimated in OTUs, while we applied the ASVs as the taxonomic unit. The more recent ASVs-based methods tend to control the amplification and sequencing error inherent of 16S rDNA data, providing a high resolution of the microbial community, down to the level of single nucleotide differences in the region of the sequenced gene [28, 29]. In contrast, the OTU-based approaches classify the sequences by grouping them based on an identity level, most commonly 97%. This strategy may generate an overestimation of the microbiota dataset caused by the misidentification of those reads derived from sequencing or PCR errors that lead to an incorrect clustering [30]. Thus, the higher number of OTUs identified in previous sugarcane microbiome studies compared to the amount of different ASV found in this work may occur due to many spurious OTUs that were erroneously classified as bacterial taxonomic groups. ASV-based methods tend to replace OTUs as the standard unit for microbiome analyses [30].

To verify if the experimental variations were comparable among the biological replicates, we evaluated the shape and completeness of the microbial abundance distribution. Non-truncated bell curve was observed in the three replicates, close to a normal distribution. Our denoised data generated an accurate and well-modeled distribution (Supplementary Fig. S3 – Additional file 1) [31]. In the heat-map graph showing the profiles of the top 25 most abundant ASVs, a similar pattern is observed across all the three replicates (Supplementary Fig. S4 – Additional file 1) supporting our data is comparable between the biological experiments. We generated an abundance-based proper rarefaction curve to get additional information on our data’s overview quality. The graphs showed a curve flatted off in bulk soil, epiphytes, and root endophytes (Supplementary Fig. S5 – Additional file 1). The sequencing approach extensively evaluated the number of microbial individuals, indicating that these data’s richness and evenness indices are comparable. However, plateaus of the number of microbial ASVs are less frequently achieved in samples that represent the endophytic communities in leaves and stems (Supplementary Fig. S5 – Additional file 1), reflecting that the sequencing depth on these samples was not sufficient to inform most of the microbial community.

### Assessment of bacterial composition of the *Saccharum* ssp.

**Fig. 1:**
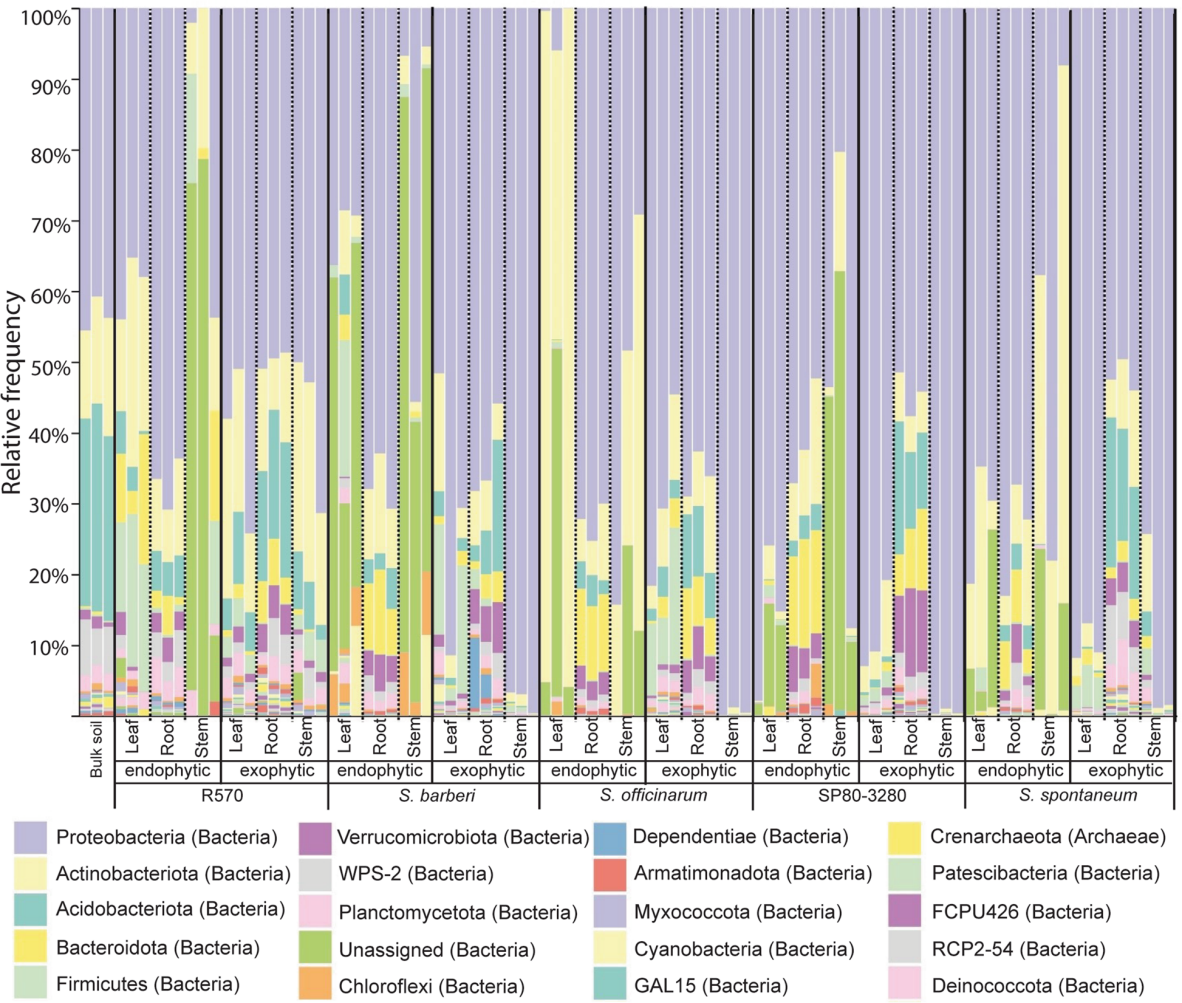
Stacked bar graphs of ASVs derived from V3/V4 16S rRNA gene sequences at the phylum level. The y-axis represents the normalized mean relative abundance of the microbial sequences. The data shows the distribution of five varieties of sugarcane (R570, *S. barberi*, *S. officinarum*, SP80–3280, and *S. spontaneum*) in endo- and exophytic compartments in the three plant organs (leaves, roots, and stems). For comparison, the bulk soil is shown on the left side of the graph. The three columns for each sample correspond to three biological replicates

The taxonomic assignment of the ASVs was performed to assess the microbial community in the *Saccharum* genotypes. Altogether the bacterial profiles within each compartment of the five cane types had a distinct pattern when compared to bulk soil, with the largest difference found in aerial parts (Fig. 1). Regarding the bacterial composition of the communities inhabiting the endophytic and the exophytic compartments, it is possible to observe differences among all genotypes (Fig. 1). These results sustain the hypothesis that host-derived factors may drive microbial colonization, filtering the taxa that inhabit the internal and external parts of the plants.

At the phylum level, most of the samples had Proteobacteria as the dominant group, followed by Actinobacteria and Acidobacteria (Fig. 1). The wild relative *S. spontaneum* presented a distinct pattern compared to those found in cultivated canes (Fig. 1). The two species, *S. barberi* and *S. officinarum*, and the hybrid SP80–3280 showed similar bacterial composition for endophytes and exophytes while the exophytic microbial community of the hybrid R570 stem has a greater phyla diversity and a more even distribution (Fig. 1). Besides, the profile of the leaf-inhabited endophytes of *S. officinarum* has the dominance of Actinobacteriota and an unassigned phylum (Fig. 1). Another singularity was the microbial community from the rhizosphere of *S. barberi,* which was dominated by the Chloroflexi phylum (Fig. 1).

**Fig. 2:**
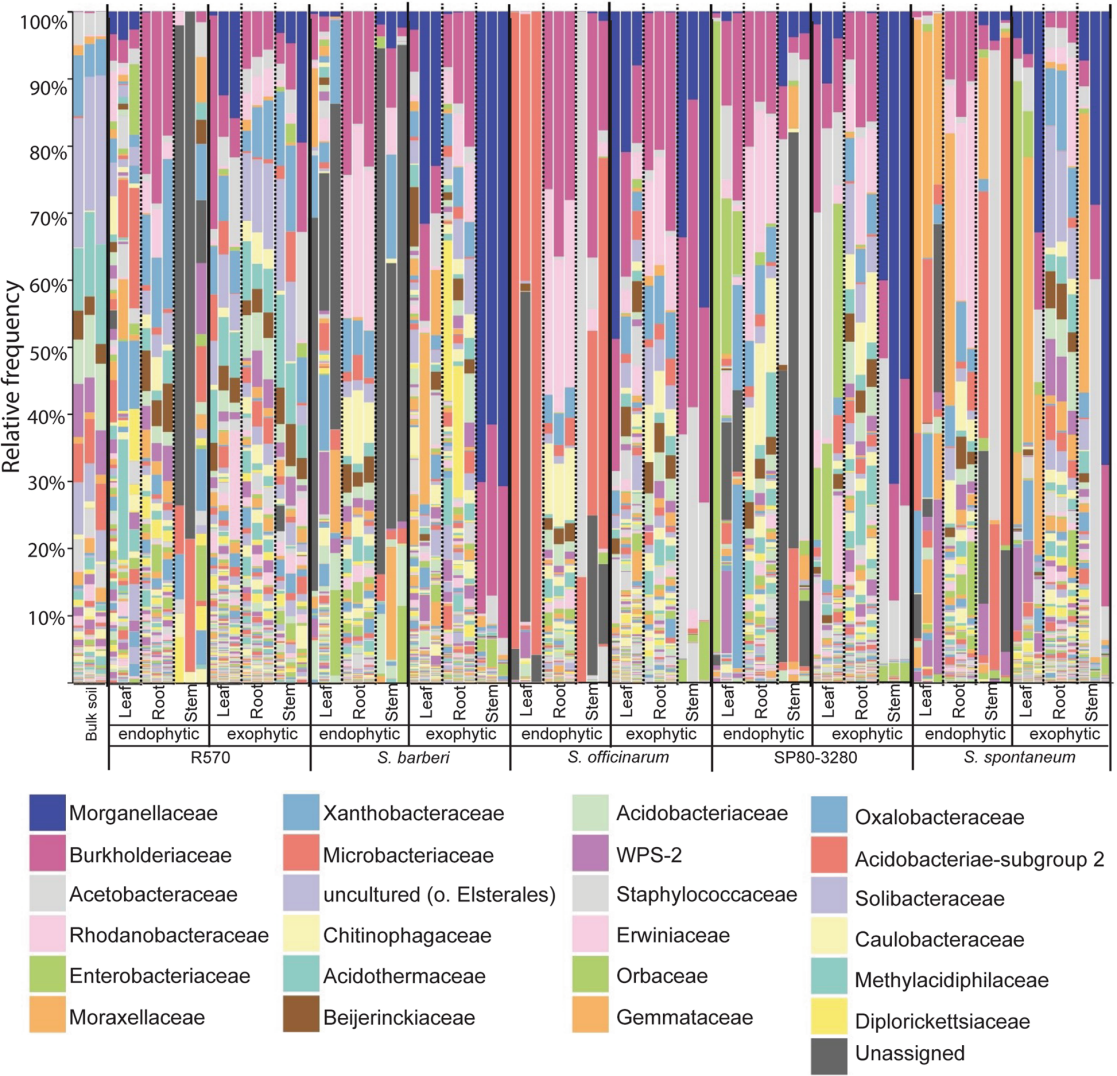
Stacked bar graphs of ASVs derived from V3/V4 16S rRNA gene sequences at the family level. The y-axis represents the normalized mean relative abundance of the microbial sequences. The data shows the distribution of five varieties of sugarcane (R570, *S. barberi*, *S. officinarum*, SP80–3280, and *S. spontaneum*) in endo- and exophytic compartments in the three plant organs (leaves, roots, and stems). For comparison, the bulk soil is shown on the left side of the graph. The three columns for each sample correspond to three biological replicates

At the family level, predominance was Morganellaceae, followed by Bulkholderiaceae, Acetobacteraceae, Rhododanobacteraceae, Enterobacteriaceae, Moracellaceae and Xanthobacteraceae, and Microbactericeae (Fig. 2). The Morganellaceae group is dominant in the samples extracted from the exophytic stem (Fig. 2), whereas within the root system the Bulkholderiaceae and Rhodanobacteraceae were more represented in relative frequency (Fig. 2). The profiles of communities from the root surface and bulk soil shared similarities among each other, with a higher number of microbial families and more evenness among them (Figs. 1 and 2). The endophytic communities from *S. officinarum* leaves and stems are predominantly composed by Acidobacteria at the phylum level (Fig. 1) and Microbacteriaceae at the family level (Fig. 2) revealing a distinct pattern to the other plants. SP80–3280 aerial parts microbial communities displayed a prevalence of Enterobacteriaceae and Acetobacteracease (Fig. 2). The profile of communities living inside and on the surface of *S. spontaneum* leaves exhibited a majority of the Moraxellaceae family (Fig. 2). Unclassified groups were more often assigned in the stem-inhabited endophytic community of R570, SP80–3280, and *S. barberi* varieties (Figs. 1 and 2), indicating the presence of little-studied organisms. The phylogenetic data disclosed that the microbial profiles differed among plant types and plant organs.

**Fig. 3:**
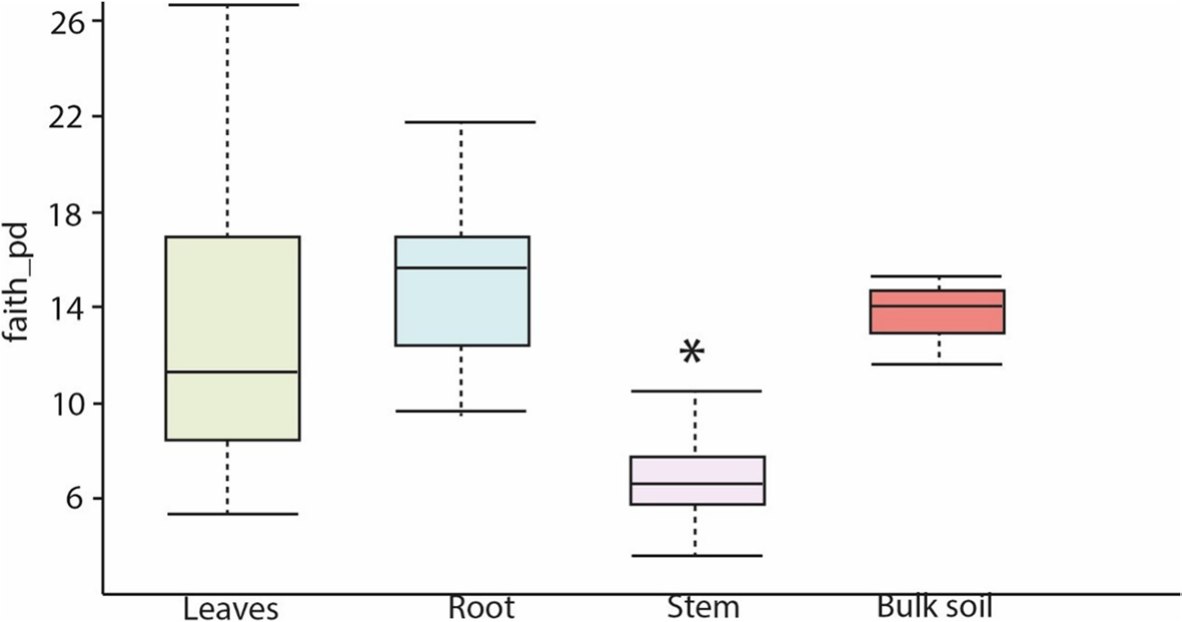
Boxplot of Alpha-diversity indices. The y-axis corresponds to Faith’s phylogenetic diversity of microbial communities sampled from sugarcane leaves (*n* = 30), roots (*n* = 30), stems (*n* = 30) and bulk soil (*n* = 3) of five varieties of sugarcane (R570, *S. barberi*, *S. officinarum*, SP80–3280, and *S. spontaneum*). The asterisk corresponds to the significant difference (*p*-value lower than 5.4 × 10^− 10^) applying comparison based on the Kruskal-Wallis methodology among all the groups

The alpha diversity indices (Fig. 3) confirmed the observed patterns of microbial taxonomic distributions observed in Figs. 1 and 2. The lowest alpha diversity is observed in stem samples (Fig. 3), the pairwise comparison based on the Kruskal-Wallis test showed that diversity indices among leaves, root, and stem significantly differ from each other (Table 1). However, there was no statistical differences in alpha diversity between the root and bulk soil (Table 1), which was expected because the root system is the primary site of interaction between plants and the soil microorganisms, increasing the probability of exchanging microorganisms compared to other plant organs.

**Table 1:** Pairwise comparison (Kruskal-Wallis test) of diversity indices communities among microbial sampled from bulk soil and sugarcane leaves, roots, and stems

Group 1	Group 2	H	***p***-value
Leaves (*n* = 30)	Root (*n* = 30)	3.9	4.93E-02
	Stem (*n* = 30)	22.0	2.78E-06
	bulk_soil (*n* = 3)	0.3	5.73E-01
Root (*n* = 30)	Stem (*n* = 30)	40.8	1.69E-10
	bulk_soil (*n* = 3)	0.8	3.81E-01
Stem (*n* = 30)	bulk_soil (*n* = 3)	7.3	7.09E-03

H: Value generated by the Kustal-Wallis test that meanings the probability of obtaining a particular number by chance

### Influence of host genetics on *Saccharum* ssp. microbiome

**Fig. 4:**
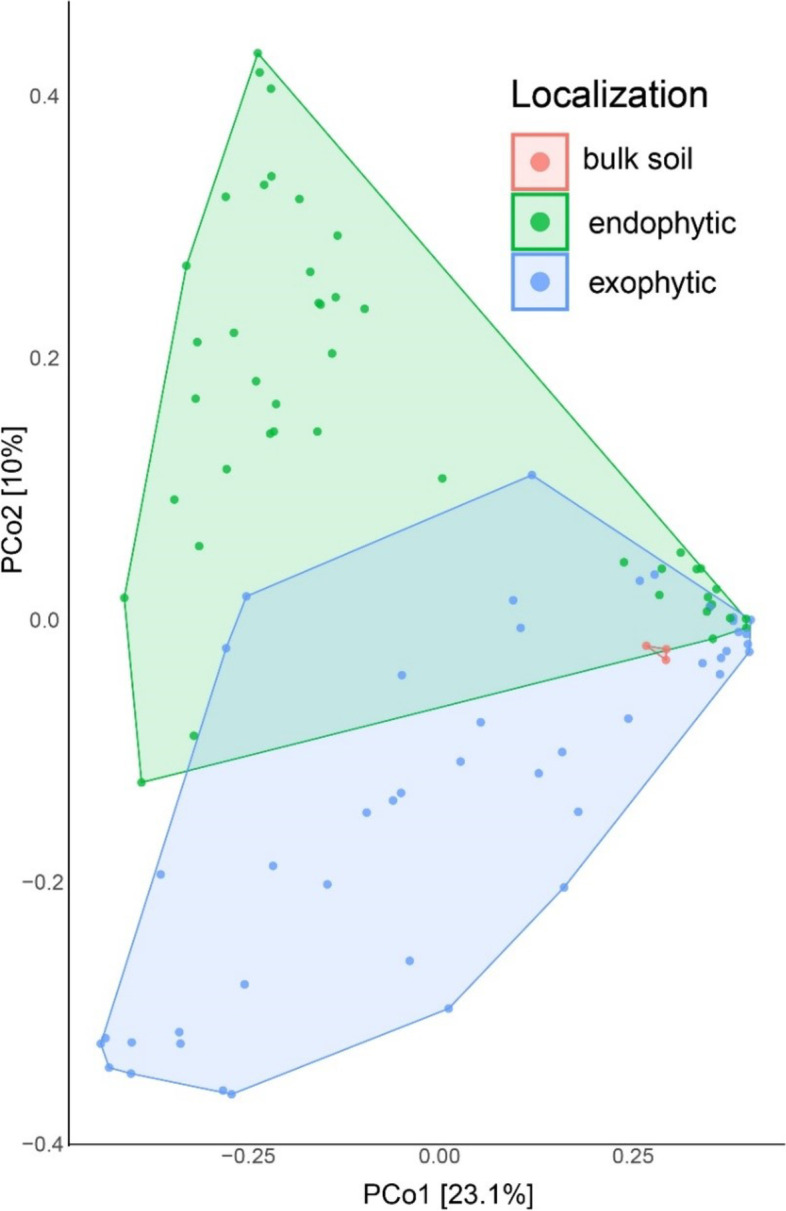
Principal coordinates analysis (PCoA) showing two-dimensional ordination of sugarcane microbiome from endo- exophytic compartments and nonplanted soil (bulk). The plot is based on an unweighted unifraction distance. The dots are the analyzed samples. The colors represent the categorization of whether the residents were collected in endophytic (light green) or exophytic (light blue) compartments, the samples referring to bulk soil are shown in red. Axes indicate the percentage of variation in the data. The samples are microbial communities collected from exo- or endophytic domains of five varieties (*S. spontaneum*, *S.barberi*, *S.officinarum*, and the hybrids R570 and SP80–3280) in three tissues (roots, stems, and leaves) and bulk soil (control)

The principal coordinates analyses (PCoA) was applied to contrast and compare our sequencing datasets. The endo- and exophytic residents were grouped in distant clusters with little overlap (Fig. 4). The spatial distance reveals a significant variation among the sample types, indicating that the structure of exophytic communities differs from that found in endophytic compartments. This data suggests that the different environmental conditions directly affect microbial communities (Fig. 4). In contrast, the corresponding dots of bulk soil were more compact, indicating that the variation of microbial communities did not differ much from each other (Fig. 4).

**Fig. 5:**
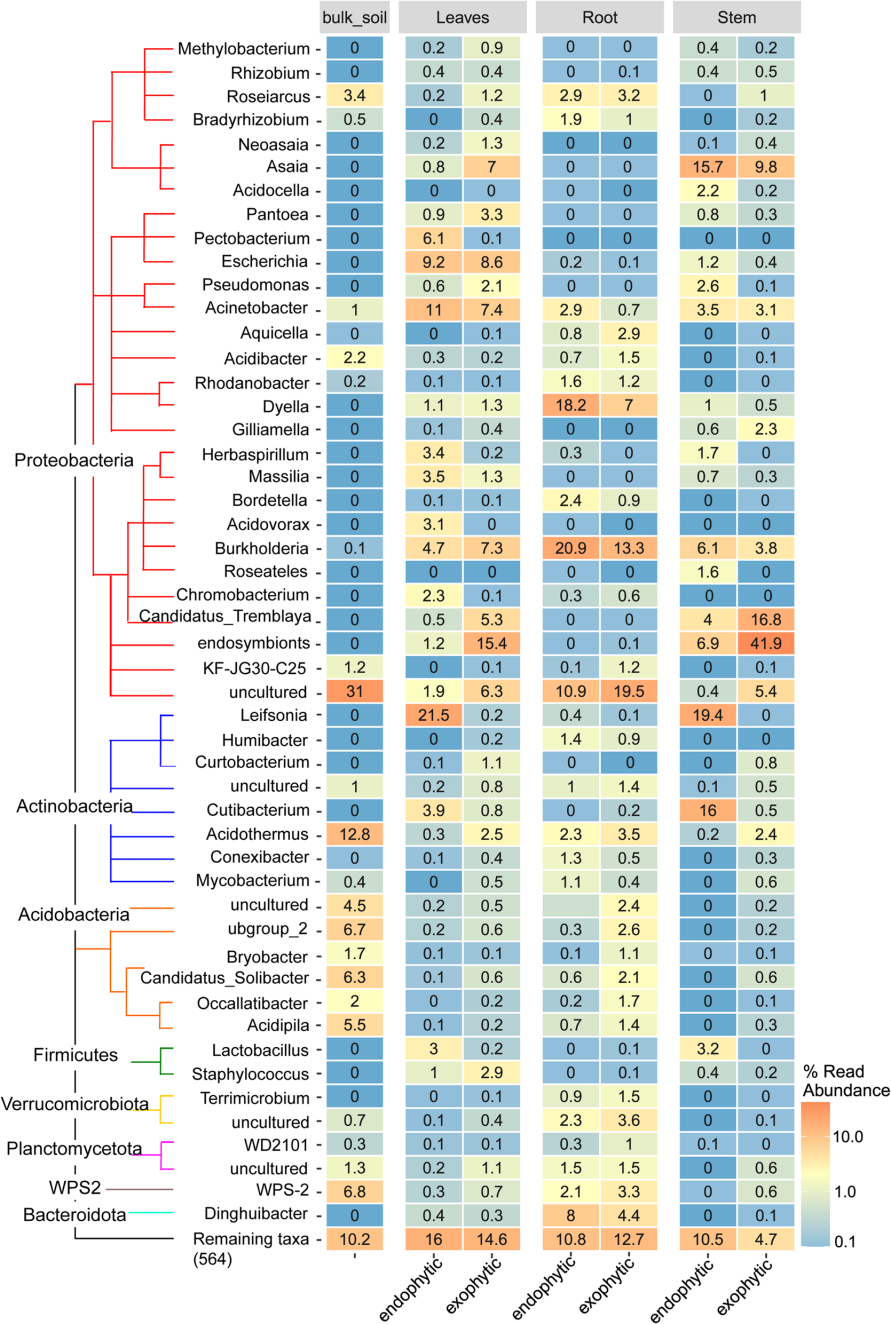
Heatmap of relative abundance of microbial taxa in exo- and endophytic compartments. The graph shows the top 25 ranked ASVs. The ASVs are grouped following their classification by phylogeny. The node of each clade represents the phylum. The genera of each ASVs are shown on the left (rows). The columns represent whether the residents were collected from endophytic or exophytic compartments. The bulk soil data are presented in a separate column on the left. Color code represents the abundance of each taxonomic group, where the warm and cold colors indicate more and less abundant, respectively. The percentages of reads abundance are displayed inside of each box

The heatmap representing the changes in the abundance of the top 25 taxa contributed to the contrast between endo- and exo- groups (Fig. 5). Interestingly, *Leifsonia* and *Herbaspirillum* ssp. displayed higher abundance in aerial endophytic compartments and low levels in roots and exophytic regions (Fig. 5). For *Leifsonia* the relative abundance was 19.4% (leaves) and 21.5% (stem), and *Herbaspirillum* ssp. 3.4% (leaves) and 1.7% (stem), respectively (Fig. 5). Both bacteria showed a relative abundance lower than 0.4% in roots (Fig. 5). Among the highly abundant bacteria living outside of the plant tissues, our data pointed to bacteria identified as endosymbiont proteobacteria (Figs. 2 and 5) of the Morganellaceae. This family includes species from the genus *Arsenophonus* which is an insect-associated endosymbiotic bacterium. Similarly, insect endophyte *Candidatus tremblaya* preferentially occupy the exterior of the aboveground plant parts, with relative abundance in 16.8 and 5.3% on the surface of stem and leaves, respectively (Fig. 5). This data may point to the presence of insects in the environment that were visiting the sugarcane leaves since the plants were grown under nonsterile conditions.

**Fig. 6:**
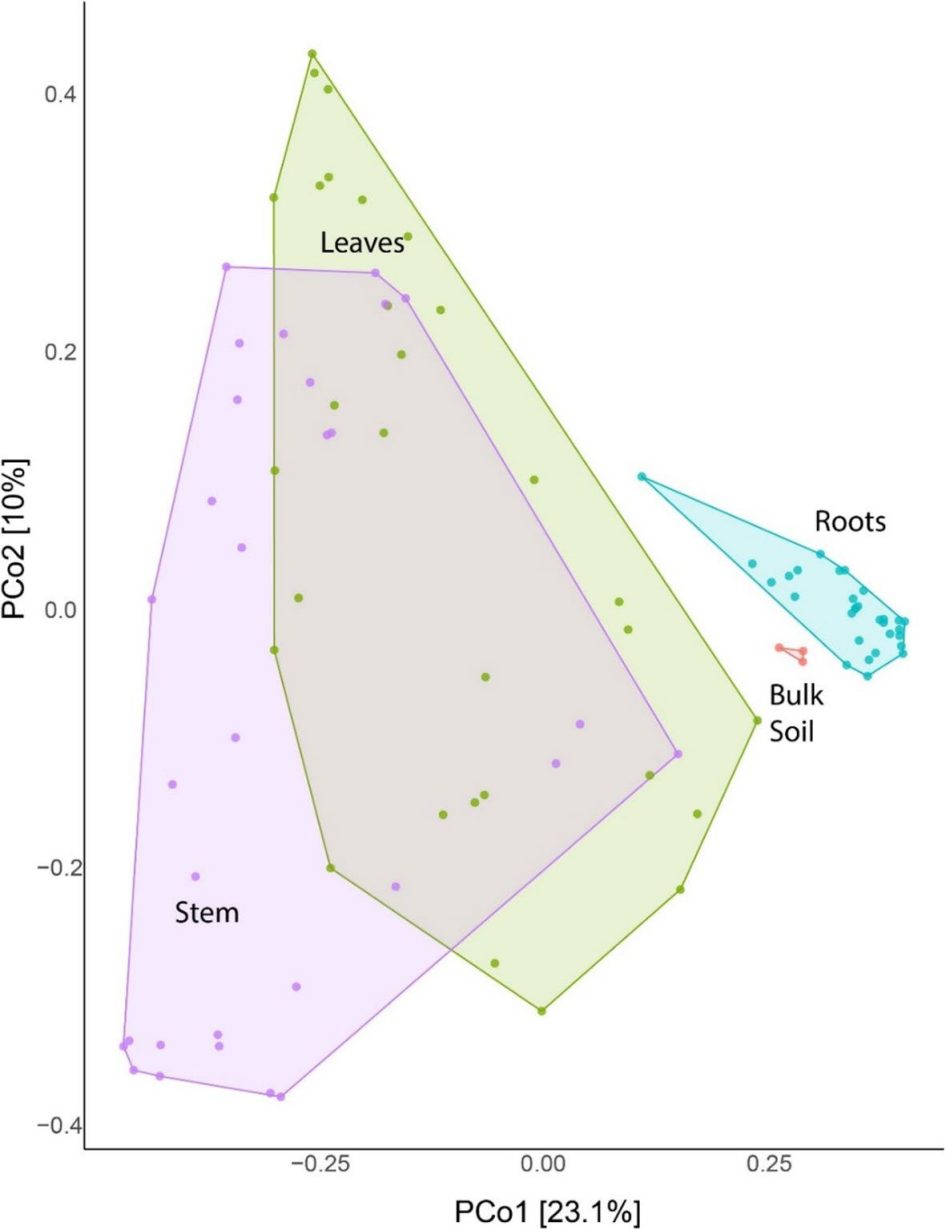
Principal coordinates analysis (PCoA) showing two-dimensional ordination of sugarcane microbiome from different organs. The plot is based on an unweighted unifraction distance. The dots are the analyzed samples. The colors represent the categorization of residents following the organ where they were collected: stem (purple), leaves (green), root (blue), and the bulk soil are shown in red. Axes indicate the percentage of variation in the data. The samples are microbial communities collected from Exo- or endophytic domains of five varieties (*S. spontaneum*, *S.barberi*, *S.officinarum*, and the hybrids R570 and SP80–3280) in three tissues (roots, stems, and leaves) and bulk soil (control)

**Fig. 7:**
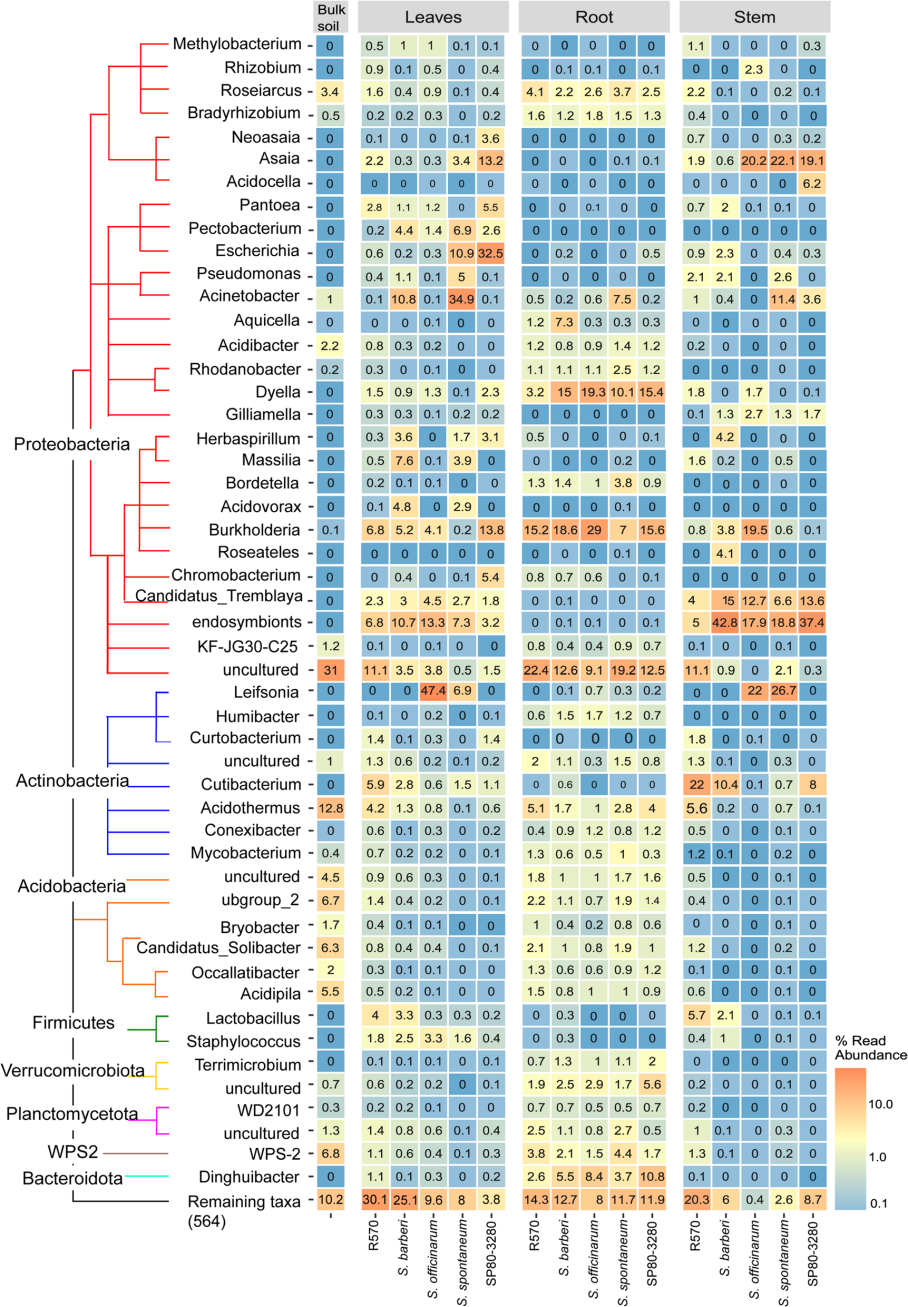
Heatmap of relative abundance of microbial taxa in different plant organs. The graph shows the top 25 ranked ASVs. The ASVs are grouped following their classification by phylogeny. The node of each clade represents the phylum. The genera of each ASVs are shown on the left (rows). The columns represent whether the residents were collected from leaves, roots, and stems. The bulk soil data are presented in a separate column on the left. Color code represents the abundance of each taxonomic group, where the warm and cold colors indicate more and less abundant, respectively. The percentages of reads abundance are displayed inside of each box

Also, we performed the PCoA comparisons between the different plant organs. The results support that there were similarities between stem and leave microbial communities. In contrast, the root and bulk soil residents have grouped apart from the other two (Fig. 6), revealing a more significant variation in community structure when comparing the root niches of the five cane genotypes with bulk soil. The top 25 most abundant taxa members responsible for these differences are shown in Fig. 7, discriminating by plant variety and organs. The proteobacteria *Neoasaia. Asaia* and *Chromobacterium* were found mainly in the leaves of SP80–3280, while the *Acidovorax, Massilia, Pectobacterium, Acinetobacter,* and *Pseudomonas* were detected in leaves of *S. barberi* and *S. spontaneum* (Fig. 7)*.* The *Aquicella* ssp. was more efficient in colonizing *S. barberi* roots than the other genotypes. *Pantoea* ssp. preferentially inhabited SP80–3280 and R570 hybrid cultivars, while an uncultured Verrucomicrobiota is prevalent in SP80–3280 root while *Lactobacillus* ssp. is numerous in the stem and leaves of R570 and *S. barberi* (Fig. 7). *Shigella* ssp. is found enriched in leaves of *S. spontaneum* and SP80–3280.

### Core microbiome for Saccharum genus

**Fig. 8:**
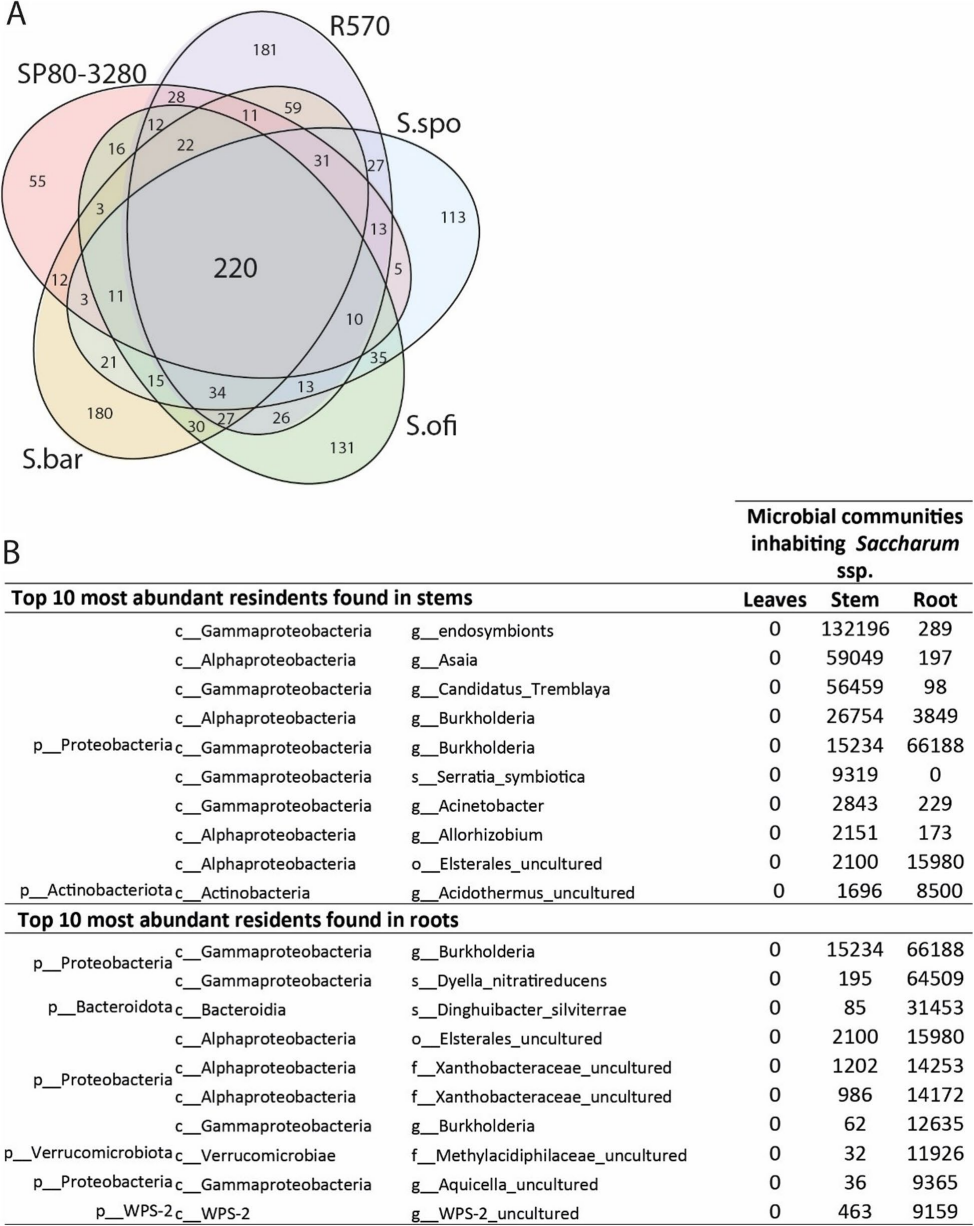
Presence of the eight highly persistent bacteria in the sugarcane core microbiome. **A** Venn diagram of microbial genera inhabiting all the five *Saccharum* varieties (SP80–3280, R570, *S. officinarum* (S.ofi), *S. spontaneum* (S.spo), and *S. barberi* (S.bar)). Numbers inside the areas represent the number of ASVs that are part of the core microbiome, and numbers in the intersections represent the number of ASVs in common. B Top 10 of the most abundant microbial groups found in stem and roots persistently inhabiting all the varieties

To identify the potential core microorganisms that inhabit *Saccharum* species, we set the microbiome framework in common for all the five cane varieties (Fig. 8 a). The genotype-specific ASVs are higher in R570 and *S. barberi*, with 181 and 180, respectively. The lowest number of unique ASVs are found in SP80–3280 with 55 ASVs. The hybrid R570 shared 59 AVSs with *S. barberi*, whereas with *S. spontaneum*, *S. officinarum* and the commercial canes shared 27 and 26 ASVs, respectively. SP80–3280 hold 5 ASVs in common with *S. spontaneum* and 16 and 12 ASVs with *S. officinarum* and *S. barberi*, respectively. The four cultivated genotypes have 22 ASVs not found in the wild relative to *S. spontaneum*. We identified 220 ASVs shared by *Saccharum* ssp. None of these 220 core ASVs are found in leaves (Fig. 8 b). Among the most abundant bacteria common to all the genotypes are *Buckholderia* ssp., *Dyella nitratireducens*, *Dinghuibacter silviterrae*, *Candidatus tremblaya*, *Acinetobacter* ssp., *Serratia symbiotica*, and uncultured bacteria (Fig. 8 b). The most ubiquitous and higher in abundance is *Acinetobacter*, observed in all plant tissues in both endophytic and exophytic compartments (Figs. 5 and 7). Its persistence may suggest that these bacteria might have a relevant role in cane growth and development.

## Discussion

A massive assessment of the microbiome structure and function of commercial crops is needed to design customized microbiomes that improve agricultural production in an environment-friendly manner. In this study, we contribute by comparing the microbiome inhabiting five *Saccharum* genotypes, a wild relative (*S. spontaneum* IN-8458), and two cultivated sugarcane cultivars extensively used in breeding programs (*S. barberi* Chunee and *S. officinarum* Badilla), as well as two hybrid commercial cultivars (R570 and SP80–3280).

Our data analysis yielded 1372 ASVs, ten and five times lower than the OTUs reported in the previous sugarcane microbiome studies [22, 27]. Difference is due to different pipelines. The ASVs-based methods require an input of high-quality reads since they control the amplification and sequencing error inherent in 16S rDNA data to resolve the microbial community down to the level of single nucleotide differences [28, 29]. Thus, denoising methods force the discarding of many sequences, which is not the case in OTU-based clustering approaches. On the other hand, the classification by grouping the sequences based on an identity level may cause misidentifying those reads derived from sequencing or PCR errors that lead to an incorrect clustering [30]. Thus, fewer taxonomic units identified here than in previous studies may occur due to fewer high quality reads retained after the denoising filtering step. In addition, there is a possibility that the amount of OTUs is overestimated due to spurious sequences erroneously classified as bacterial taxonomic groups. The variation in biological results due to different pipelines leans to reduce, as ASV-based methods tend to replace OTUs as the standard unit for microbiome analysis [30].

Our data revealed a strong genotype-influence on the assembly of microbial communities in *Saccharum* ssp., which corroborates with previously published results, showing the key influence of the host genotype to shape the microbiome [32]. The diversity analysis disclose that the lowest alpha diversity index is found in stem samples. Similar results were found in previous sugarcane microbiome data [22]. Rather than reflecting the truth, it may be caused by the limitation of the technique, as the number of chloroplast-related reads was higher in the stem samples in both studies [22] (Supplemental Table 2). The low number of reliable reads was revealed by rarefaction curves (Supplementary Fig. 5 – Additional file 1), indicating that the sequencing depth was insufficient to access the microbial communities living the stem extensively, suggesting that the diversity numbers might be underestimated.

The assessment of microbial composition inside and outside of different plant organs across five genotypes allows us to compare genotype influences that directly impact controlling bacterial communities. Our data corroborated with the hypothesis that plant organs, either internal or external parts, actively contribute to determining the structure and the composition of resident bacteria. A strong genotype effect was detected at phylum and family levels, influencing the selection of microorganisms occupying different plant compartments (Figs. 1 and 2). The sorting effect found in sugarcane mirrors similar findings in other crops, including rice [33], poplar [34], soybean [35], maize [36].

The *S. spontaneum*, the unique wild cane included in this study, presents a profile of endophytic bacterial composition distinct from the cultivated genotypes (Figs. 1 and 2). Sugarcane microbiomes are susceptible to the surroundings and genetic influences. The most distinctive feature occurs in the aerial parts, where the absence of leaf residents in the core of 220 ASVs of the *Saccharum* genus may indicate the relevance of environmental factors in structuring the bacterial communities in this compartment.

Among the relatively known are the sugarcane endophytes *Leifsonia* and *Herbaspirillum* [37]. They were not detected in all the varieties, *Leifsonia* ssp. showed enrichment in aerial parts of the parental species *S. officinarum* and *S. spontaneum* compared to *S. barberi* and hybrid cultivars (Fig. 7). In contrast, the diazotrophic *Herbaspirillum* ssp. is prevalent in *S. barberi*, *S. spontaneum* and SP80–3280 (Fig. 7). *Leifsonia* ssp. colonizes the xylem vessels, mesophyll and bundle sheath surrounding the vascular system [37]. *Leifsonia* ssp. is considered an asymptomatic endophyte as it is often found in natural grass fields [38]. Commercial monocots, such as corn and rice have a neutral and, in some cases, beneficial role for the host [39–41]. However, in susceptible sugarcane cultivars, the strain *Leifsonia xyli* subsp. *xyli* (Lxx) causes ratoon stunting disease [42, 43]. Like *Leifsonia* ssp., *Herbaspirillum* ssp. seems to be an obligate endophyte frequently found in Gramineae [44, 45], it is confined to the vascular system especially in the protoxylem [46]. Both endophytes cannot survive in the soil, in Lxx this is explained by its largely reduced genome that lacks genes necessary for survival outside the host [47]. This characteristic may explain the low abundance of these bacteria in roots and outside of the plant found in our data (Fig. 5).

The persistence of 220 microorganisms commonly found within the cane microbiome (Fig. 8) suggests their potential symbiotic relationship with this plant. The *S. symbiotica* and *Acinetobacter* ssp. might be unexplored beneficial bacteria relevant to sugarcane. *Acinetobacter* is known for growth promotion in several plant species, such as Pearl millet [48], soybean [49], duckweed, and lettuce [50], cucumber, Chinese cabbage, and crown daisy [51]. In cane, it has already been identified in plant tissues through culture-dependent [52] and independent [27] methods. However, its biotechnological potential is poorly described [52]. *S. symbiotica* is commonly found in the aphid gut and represents an attractive model for understanding the mechanism of shaping interspecific symbioses. A tripartite interaction between the aphid *Aphis fabae*, a cultivable *S. symbiotica* bacterium, and the host plant *Vicia faba* was previously described [53], where the plant works as a vector to transfer the symbiont to uncolonized aphids. *S. symbiotica* acts as a beneficial resident for the insects by providing metabolic supplements [54], heat tolerance [55] parasitoid resistance [56], while for plants it promotes the root system growth [53]. The ubiquitous presence of aphid-endophytic bacteria may indicate that a similar complex ecological interaction may also occur in cane species.

In summary, the high-input sequencing approach revealed that the microbiome is a valuable resource for improving agricultural practices. One strategy is the application of a customized microbial consortium to improve agricultural yields. However, in order to select the beneficial microorganisms, knowledge on the major players linked to the desired traits is needed. Our data provide a core of 220 microorganisms resident of five cane genotypes, their persistence might be associated with a yet unknown biological function in the plant. Among those, *S. symbiotica* and *Acinetobacter* ssp. may represent unexplored beneficial bacteria. They are promising candidates for plant-microbe interaction studies. Additionally, we revealed that the environment and genetic factors mold the microbial structure in cane tissues. It remains to be seen which aspect has the most significant impact on the microbial selection in sugarcane fields.

## Methods

### Plant material and soil sampling

*Saccharum barberi* (Chunee), *Saccharum officinarum* (Badilla de Java), *Saccharum spontaneum* (IN-8458), the sugarcane hybrid commercial cultivars R570 and SP80–3280 were obtained from the varietal garden of Sugarcane Research Center - Agronomic Institute of Campinas (IAC) located in Ribeirão Preto- SP, Brazil. Nodes segmented stalks to isolate the potential buds are kept in trays with moistened vermiculite for 33 days. The segmented nodes with emerging plantlets were transferred to vases with soil and grown in the greenhouse from October 12, 2018 to January 12, 2019. The soil was collected at two points 10 m apart from one another in the Atlantic Rain Forest reserve of the University of São Paulo, campus Armando de Salles de Oliveira (23°33′58.16″ S 46°43′44.586″ W). The collected soil was mixed and distributed into 20 plots with 25 l of soil and maintained under a controlled regular watering. We cultivated one plant per vase for 3 months. Tissues were collected from three healthy plants (Supplemental Fig. S1 – Additional file 1). The 4–5 youngest fully expanded leaves, the stem, and root systems were sampled (Supplemental Fig. S2 – Additional file 1). For control, three plots were kept without plants and it is referred to here as bulk soil (Supplemental Fig. S1 – Additional file 1).

### Microbial sample collection

Microbial communities were collected from inner and external parts of 4–5 youngest and fully expanded leaves, stalks, and whole root systems. The organs were washed as previously described [22]. Briefly, sugarcane organs were individualized and placed in trays once cleaned with 70% ethanol. Each tissue was processed independently, it was manually washed at least twice in ice-cold PBS-tween20 (7 mM Na2HPO4, 3 mM NaH2PO4, pH 7.0 and 0.05% (v/v) tween20) solution. The resulting suspension was seeped through a filter paper and kept in a glass bottle on ice. The flow through was centrifuged at 3000 x g for 15 min at 4 °C. The pellet was resuspended in 10 ml of PBS-tween20 and transferred to a 15 ml plastic tube for the second round of centrifugation under the same condition. The supernatant was discarded and the resulting precipitate was defined as an exophytic microbial community. The washed organs were transferred to a plastic bag containing enough ice-cold distilled water to cover the tissues. The samples were sonicated using an ultrasonic bath (Thornton – Inspec electronica) for 5 minutes. The material was placed on filter paper to remove the excess water and chopped into small pieces (~ 1 cm for root and ~ 3 cm leaf/stem). To collect the samples enriched with endophytic microorganisms, a ratio of 1:1 (v/w) of ice-cold PBS-tween20: plant tissue was maintained and mixed using a regular kitchen blender, previously UV-sterilized, washed with 70% (v/v) ethanol. The blended solution was filtered through sterilized filter paper and centrifuged at 3000×g for 15 min at 4 °C. The pellet was resuspended in 10 ml of PBS-tween20, transferred, and centrifugated in a 15 ml plastic tube. The soils were collected from unplanted plots (bulk) as a control. The samples were stored at − 20 °C. All the materials and solutions were sterilized by autoclaving unless another is specified. The experiment was performed under three biological replicates.

### DNA extraction and quantification

DNA was extracted following the instructions from the DNAeasy PowerLyzer Microbial Kit (Qiagen. ID 12255). An additional lysis step was included to improve the extraction yield, in which samples were heated at 65 °C for 10 minutes. After that, most of the root and bulk soil samples still showed a brownish color. Then, another cleanup step using HTR Reagent® (Omega Bio-Tek) from E.Z.N.A.® Soil DNA Kit (Omega Bio-Tek). Briefly, the volume was adjusted to 200 μL with water. Then, we added 100 μL of HTR Reagent, followed by an intense vortex mix, and sat at room temperature for 2 minutes. The suspension was centrifuged at maximum speed for 2 minutes at room temperature. The cleared supernatant was transferred to a new microcentrifuge tube. The samples were stored at − 20 °C.

### Library preparation and sequencing of 16S rDNA

The library was built based on a PCR amplification reaction carried out with the primer pair targeting to V3/V4 region of 16S rDNA (341-F: CCTACGGGNGGCWGCAG and 805-R: GACTACHVGGGTATCTAATCC) [53] supplemented with PNA clamps for sugarcane mitochondria and chloroplast ribosomal RNA genes using the following the instruction from the report previously published [22]. The library construction and amplicon sequencing were performed at the Animal Biotechnology Lab, Department of Animal Science (ESALQ/USP Piracicaba, Brazil) on a MiSeq platform following the manufacturer’s guidelines using the Nextera XT assay and Nextera XT V2 index kit.

### Data processing and statistical analysis

Demultiplexed sequence data in the FASTQ format was imported into the QIIME2 [57] v2020.2 version. DADA2 [26] was used to quality filter, trim, denoise, and merge the data pairs. Chimeric sequences were removed using the consensus method. The sequences flagged as non-chimeras were retained. The taxonomic assignment of the representative sequences was carried out using the feature-classifier2 plugin implemented in QIIME2 against the SILVA SSU non-redundant database (138 release). Those sequences showing > 99% identity is clustered into a single Operational Taxonomic Units (OTUs). The classifier used here was previously trained and can be found at this link https://forum.qiime2.org/t/silva-138-classifiers/13131. After the taxonomic assignment, sequences identified from chloroplast, mitochondria, and eukaryotes were removed using the command “QIIME taxa filter-table” from QIIME2. The heatmap figures, 14 rarefaction curves, and octave plots were generated by the R library ampvis2 [58], using as input the QIIME2 metadata and taxonomy table exported as biom file.

## Supplementary Information

**Additional file 1: Supplemental Figure S1.** Schematic view of the experimental design. **Supplemental Figure S2.** Harvesting photos of the samples. **Supplemental Figure S3.** Octave plots showing comparison among biological replicates. **Supplemental Figure S4.** Patterns of microbial composition profiles across biological replication. **Supplemental Figure S5.** Rarefaction curves of microbial clusters in the microbiome from different plant organs and nonplanted soil (bulk).

**Additional file 2: Supplemental Table S1.** Table of the total number of trimmed reads and percent alignment to chloroplast and mitochondria genomes. **Supplemental Table S2.** Table of the total number of filtered, merged, and non-chimeric sequences by DADA2 denoise software.

## Data Availability

Data availability Raw data was submitted to NCBI under the BioProject code PRJNA604994 and Submission ID of SUB6926885. Plant material Plant material used in this work is provided by Dr. Silvana Creste from the varietal garden of Sugarcane Research Center - Agronomic Institute of Campinas (IAC) located in Ribeirão Preto- SP, Brazil and is part of the Plant Breeding Collection.

## Abbreviation

PNA: Peptide Nucleic Acid
