# Supplementary material for: Towards defining the core Saccharum microbiome: input from five genotypes
Source: BMC Microbiol. 2022 Aug 8;22:193. doi: 10.1186/s12866-022-02598-8 (PMC9358853; doi:10.1186/s12866-022-02598-8)
Supplement: Supplementary file 1 — Additional file 1: Supplemental Figure S1. Schematic view of the experimental design. Supplemental Figure S2. Harvesting photos of the samples. Supplemental Figure S3. Octave plots showing comparison among biological replicates. Supplemental Figure S4. Patterns of microbial composition profiles across biological replication. Supplemental Figure S5. Rarefaction curves of microbial clusters in the microbiome from different plant organs and nonplanted soil (bulk). [file 12866_2022_2598_MOESM1_ESM.pdf]

Additional file 1: Supplemental figures.

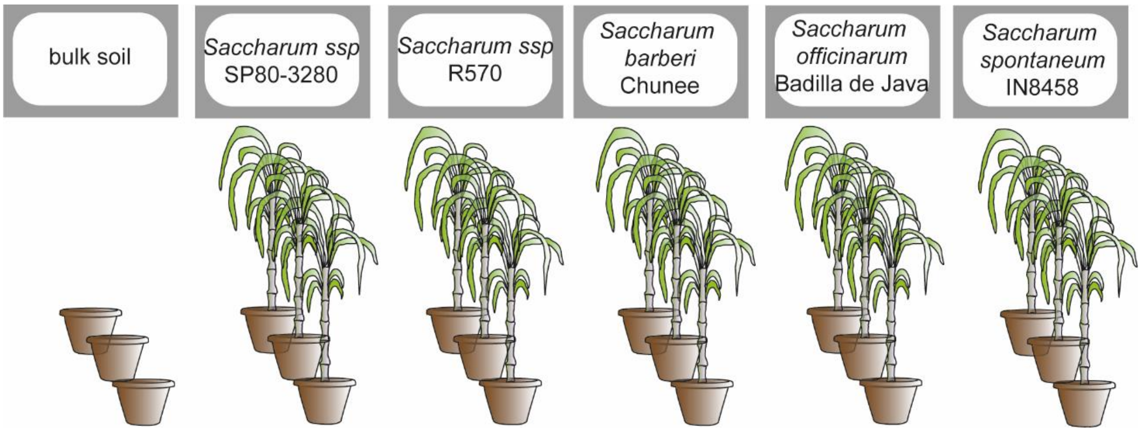

Supplemental figures S1 - Schematic view of the experimental design. Soil collected from the Atlantic Rain Forest reserve was distributed into 20 plots containing about 25 liters each. The 1-month-segmented sugarcane nodes were transferred to the soil and kept growing for 3 months under a regular watering system. Three plots of each sugarcane species (*S. barbari* - Chunee, *S. officinarum* – Badilla de Java, and *S. spontaneum* – IN8458) and two hybrid commercial cultivars (SP80-3280 and R570) were randomly placed in the greenhouse. As control plots with non-cultivated soil (bulk soil) were maintained.

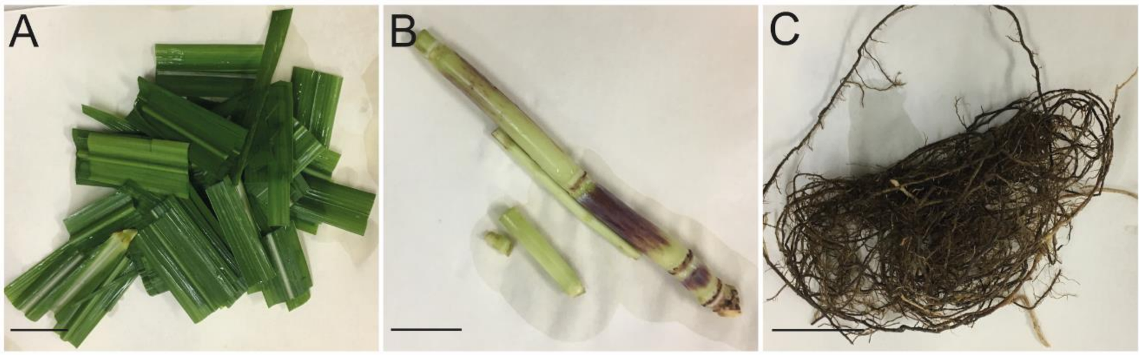

Supplemental figures S2 - Harvesting photos of the samples. Representative photo of SP80-3280 sugarcane. The external microbiomes were removed with manual washing and immersed in the tissues in a cold solution of PBS added 0.05% (v / v) Tween20. The internal tissue microbiome, on the other hand, was obtained from previously washed tissues (A to C). These were crushed using a standard kitchen mixer in a cold solution of PBS added 0.05% (v / v) Tween20. (A) leaves, (B) stem, and (C) roots. Bars correspond to 5 cm.

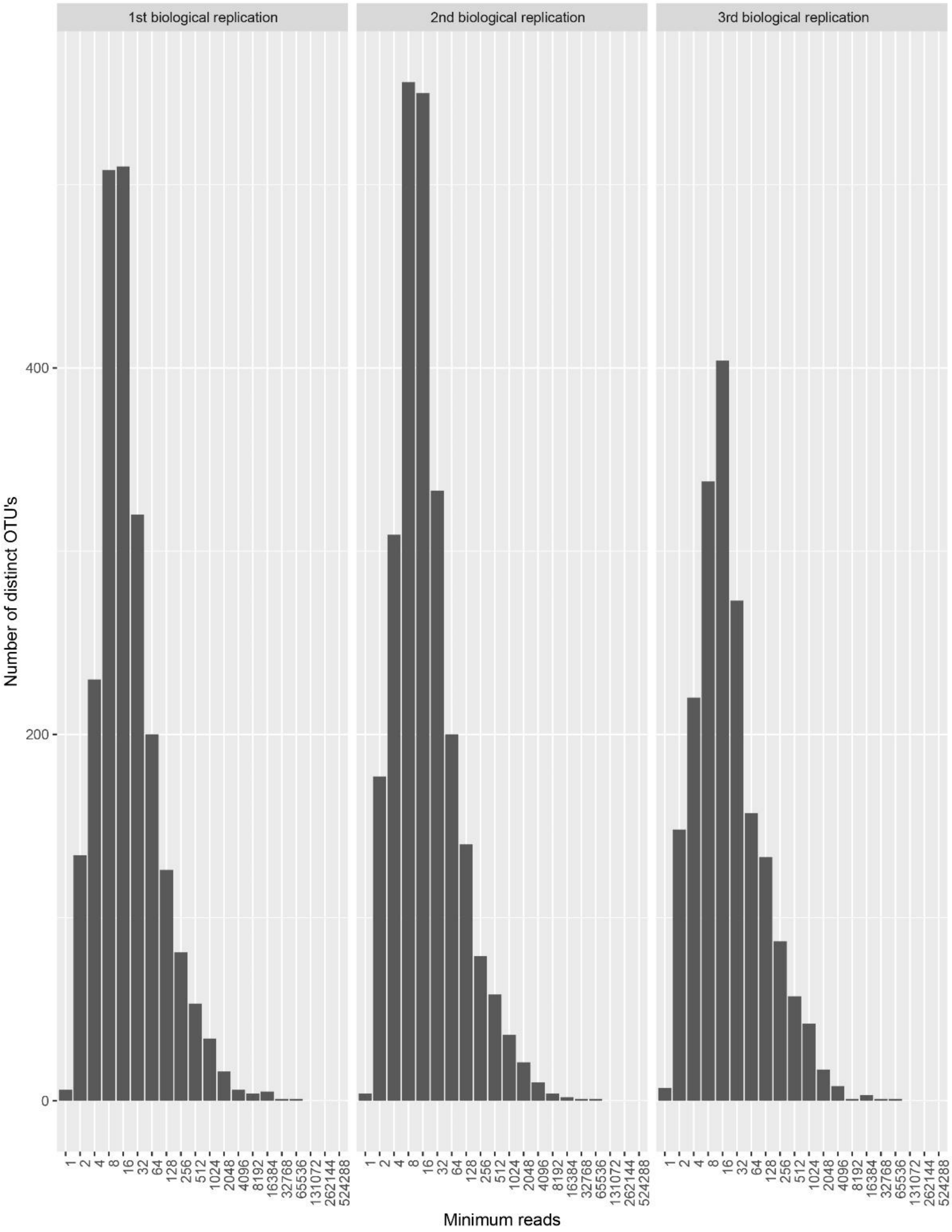

Supplemental figures S3 - Octave plots showing comparison among biological replicates. The panel shows the histograms generated based on the microbial abundance distribution of OTUs by the read depth on a logarithmic scale with base 2. The distribution was obtained from the subsets of the reads after submitting the raw reads to the dada2 denoise pipeline. The log-series of the three biological replications are compared.

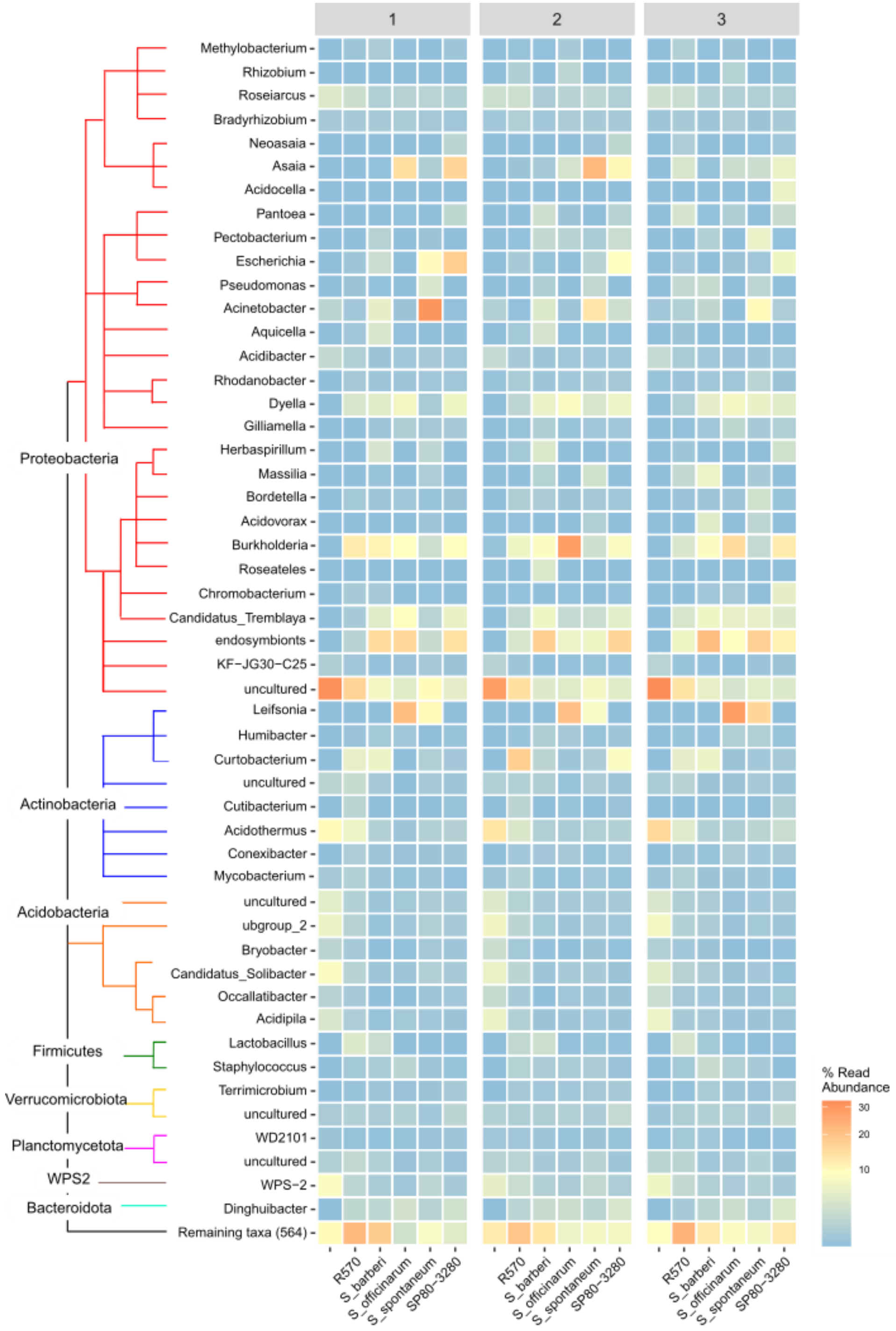

Supplemental figures S4 – Patterns of microbial composition profiles across biological replication. The heatmap shows the top 25 ranked ASVs. The ASVs are grouped following their classification by phylogeny, the phylum is shown at the node of each clade, and the genera of each ASVs are shown on the left (rows). The three columns represent each biological repetition. The gradient colors represent the abundance of microbial communities, where the warm and cold colors indicate more and less abundance, respectively.

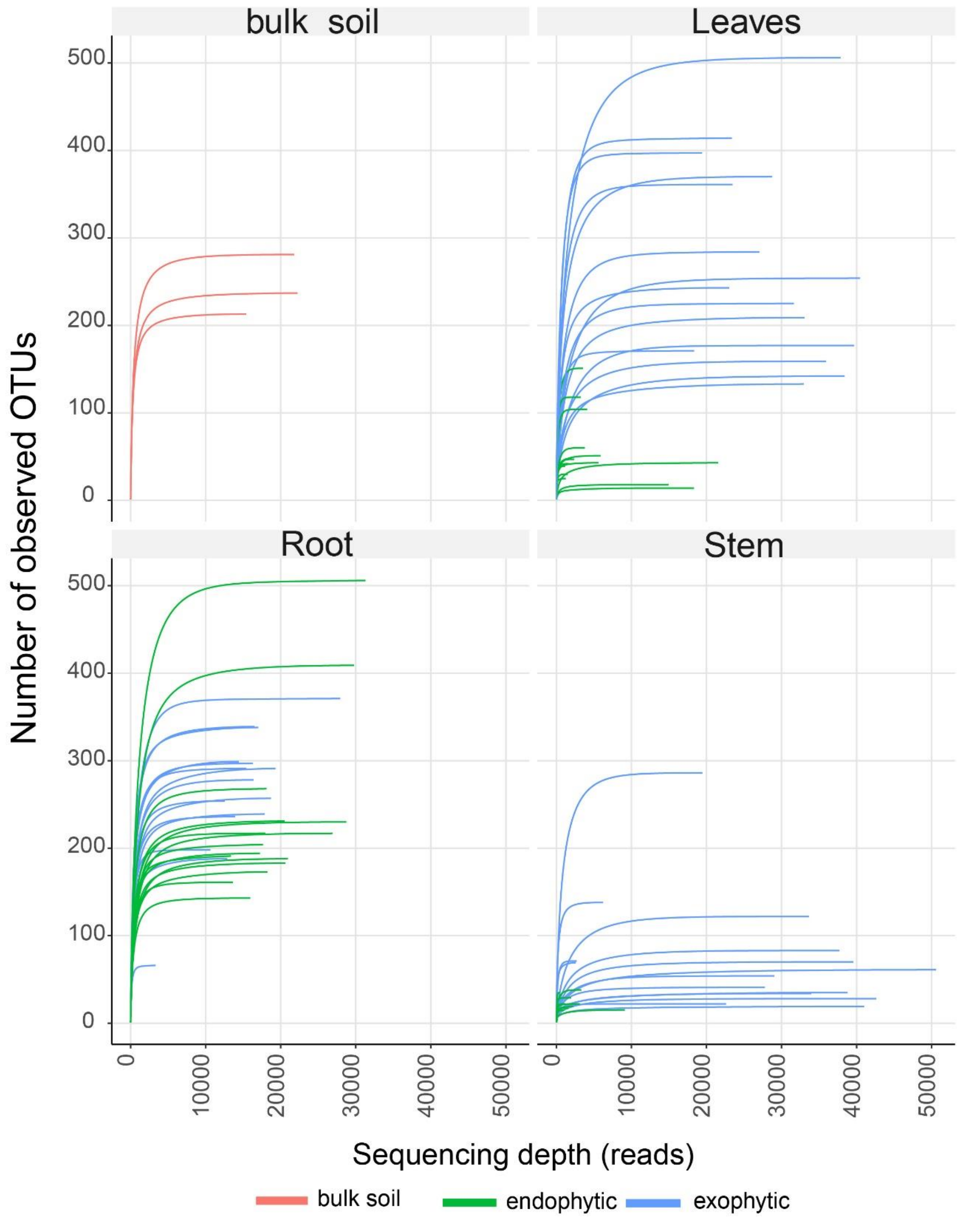

Supplemental figures S5 – Rarefaction curves of microbial clusters in the microbiome from different plant organs and nonplanted soil (bulk). Comparison of the data collected from endophytic (green) and exophytic (blue) compartments, and bulk soil (red). The y-axis represents the number of observed microbial taxa and the x-axis the sequencing depth.
