# Supplementary material for: Towards defining the core Saccharum microbiome: input from five genotypes
Source: BMC Microbiol. 2022 Aug 8;22:193. doi: 10.1186/s12866-022-02598-8 (PMC9358853; doi:10.1186/s12866-022-02598-8)
Supplement: Supplementary file 2 — Additional file 2: Supplemental Table S1. Table of the total number of trimmed reads and percent alignment to chloroplast and mitochondria genomes. Supplemental Table S2. Table of the total number of filtered, merged, and non-chimeric sequences by DADA2 denoise software. [file 12866_2022_2598_MOESM2_ESM.pdf]

Supplemental Table S1 – Total number of trimmed reads and percent alignment to chloroplast and mitochondria genomes.

		Chloroplast				Mitochondria			
		Aligned reads				Aligned reads			
		forward		reverse		forward		reverse	
Leaves	Endo	244480	(17.7%)	223941	(18.1%)	804478	(58.2%)	634143	(51.3%)
	Exo	18279	(1.4%)	16957	(1.4%)	87100	(6.7%)	151996	(12.9%)
Stem	Endo	216685	(12.0%)	198996	(12.4%)	1449544	(80.2%)	1214015	(75.7%)
	Exo	34722	(2.3%)	33616	(2.4%)	426106	(28.1%)	375094	(26.8%)
Root	Endo	868	(0.1%)	1186	(0.1%)	108640	(10.5%)	24483	(2.5%)
	Exo	1719	(0.2%)	1895	(0.2%)	5645	(0.6%)	3820	(0.5%)
bulk soil		1040	(0.4%)	1049	(0.5%)	406	(0.2%)	238	(0.1%)

Supplemental Table S2 – Total number of filtered, merged, and non-chimeric sequences by DADA2 denoise software.

		input sequences	filtered sequences	percentage of input passed the filter	merged	percentage of merged sequences	non-chimeric sequences	percentage of input non-chimeric
Leaves	Endo	1667666	646315	38.8%	588565	35.3%	535149	32.1%
	Exo	1385405	541294	39.1%	478064	34.5%	470594	34.0%
Stem	Endo	2017452	796841	39.5%	751176	37.2%	685153	34.0%
	Exo	1727798	730743	42.3%	689366	39.9%	651548	37.7%
Root	Endo	1236001	447073	36.2%	343321	27.8%	323264	26.2%
	Exo	1060996	369250	34.8%	241843	22.8%	234588	22.1%
bulk soil		277222	94275	34.0%	63738	23.0%	59994	21.6%
Total		9372540	3625791		3156073		2960290	
